# Cannabidiol Regulates Gene Expression in Encephalitogenic T cells Using Histone Methylation and noncoding RNA during Experimental Autoimmune Encephalomyelitis

**DOI:** 10.1038/s41598-019-52362-8

**Published:** 2019-10-31

**Authors:** Xiaoming Yang, Marpe Bam, Prakash S. Nagarkatti, Mitzi Nagarkatti

**Affiliations:** Department of Pathology, Microbiology and Immunology, School of Medicine, University of South Carolina Columbia, South Carolina, 29209 USA

**Keywords:** Long non-coding RNAs, Epigenetics in immune cells

## Abstract

Cannabidiol (CBD) has been shown by our laboratory to attenuate experimental autoimmune encephalomyelitis (EAE), an animal model of multiple sclerosis (MS). In this study, we used microarray and next generation sequencing (NGS)-based approaches to determine whether CBD would alter genome-wide histone modification and gene expression in MOG sensitized lymphocytes. We compared H3K4me3 and H3K27me3 marks in CD4+ T cells from naïve, EAE and CBD treated EAE mice by ChIP-seq. Although the overall methylation level of these two histone marks did not change significantly, the signal intensity and coverage differed in individual genes, suggesting that CBD may modulate gene expression by altering histone methylation. Further analysis showed that these histone methylation signals were differentially enriched in the binding sites of certain transcription factors, such as ZNF143 and FoxA1, suggesting that these transcription factors may play important roles in CBD mediated immune modulation. Using microarray analysis, we found that the expression pattern of many EAE-induced genes was reversed by CBD treatment which was consistent with its effect on attenuating the clinical symptoms of EAE. A unique finding of this study was that the expression of many miRNAs and lncRNAs was dramatically affected by CBD. In summary, this study demonstrates that CBD suppresses inflammation through multiple mechanisms, from histone methylation to miRNA to lncRNA.

## Introduction

Marijuana (*Cannabis sativa*) has many biologically active compounds and its medicinal value has been known for centuries. Two main active ingredients in marijuana are delta-9-tetrahydrocannabinol (THC) and cannabidiol (CBD). THC, the psychotropic component, is used for pain relief, stimulating appetite, and as an anti-emetic in cancer and HIV/AIDS patients^1^. CBD is a non-psychoactive compound in marijuana with potential efficacy against epilepsy and autoimmune diseases^2^. THC exerts its function through cannabinoid receptors, CB1 and CB2. CB1 is predominantly expressed in the brain while CB2 is primarily found on immune cells^3^. However, CBD does not bind to or activate cannabinoid receptors directly. In neuronal cells, CBD demonstrates agonist activity on 5-HT_1A_ receptor and regulates 5-HT_1A_ receptor-mediated neurotransmission^4,5^. In immune system, studies from our lab as well as those from others have shown that both THC and CBD have anti-inflammatory properties^2,6–9^. CBD has been shown to attenuate many autoimmune diseases including experimental autoimmune encephalomyelitis (EAE), autoimmune hepatitis, colitis and collagen-induced arthritis^6,8,10,11^. CBD may exert some of its effect through several receptors, including vanilloid receptor 1 (TRPV1), G-coupled receptor 55 (GPR55), and 5-HT_1A_ receptors depending on the disease model.

EAE is an animal model of multiple sclerosis (MS). MS is a neurodegenerative autoimmune disease. The disease often begins with inflammatory attacks against the white matter of the brain, producing neurological disorders including loss of sensation, lack of coordination, bowel and bladder incontinence, difficulty in walking and paralysis^12^. EAE partially mimics the immunopathological process of MS by immunizing mice with myelin-derived antigens such as myelin basic protein (MBP), proteolipid protein (PLP), myelin associated glycoprotein (MAG) and myelin oligodendrocyte glycoprotein (MOG)^13^. These immunodominant antigenic epitopes trigger acute and chronic autoimmune disease. It has been shown that in EAE models and MS patients, naïve CD4+ T cells can differentiate into pro-inflammatory Th1 and Th17 cells which cross blood-brain barrier and contribute to neuro inflammation^14–16^. On the other hand, FoxP3 + regulatory T cells (Tregs) play a critical role in the downregulation of EAE^17^. In addition, previous studies from our lab have demonstrated that CBD can also attenuate clinical symptoms of EAE by inducing immunosuppressive Myeloid-derived Suppressor Cells (MDSC)^6^.

Although THC and CBD have shown anti-inflammatory property in various disease models, the underlying mechanism is not clear. Our studies have suggested the THC and CBD may modulate histone modification and miRNA expression in immune cells. For example, THC regulates gene expression in lymphocytes by altering histone methylation and acetylation^18,19^. In this study, we investigated whether activation of T cells with MOG antigen would alter gene expression and histone methylation leading to differentiation of T cells into proinflammatory phenotype and whether CBD treatment would reverse these effects. To that end, we used microarray and next generation sequencing (NGS)-based approaches to determine whether CBD could alter histone methylation (H3K4me3 and H3K27me3), and expression of non-coding RNA (miRNA and long non coding RNA (lncRNA)) in CD4+ T lymphocytes from naïve, MOG + vehicle and MOG + CBD treated mice. Our results suggested that histone methylation as well as non coding RNAs may play important roles in inflammatory T cell development and that CBD-mediated immune modulation may result from restoration of such alterations.

## Results

### Effect of CBD on genome-wide histone H3K4me3 and H3K27me3 methylation in splenic CD4+ T cells

**Figure 1:**
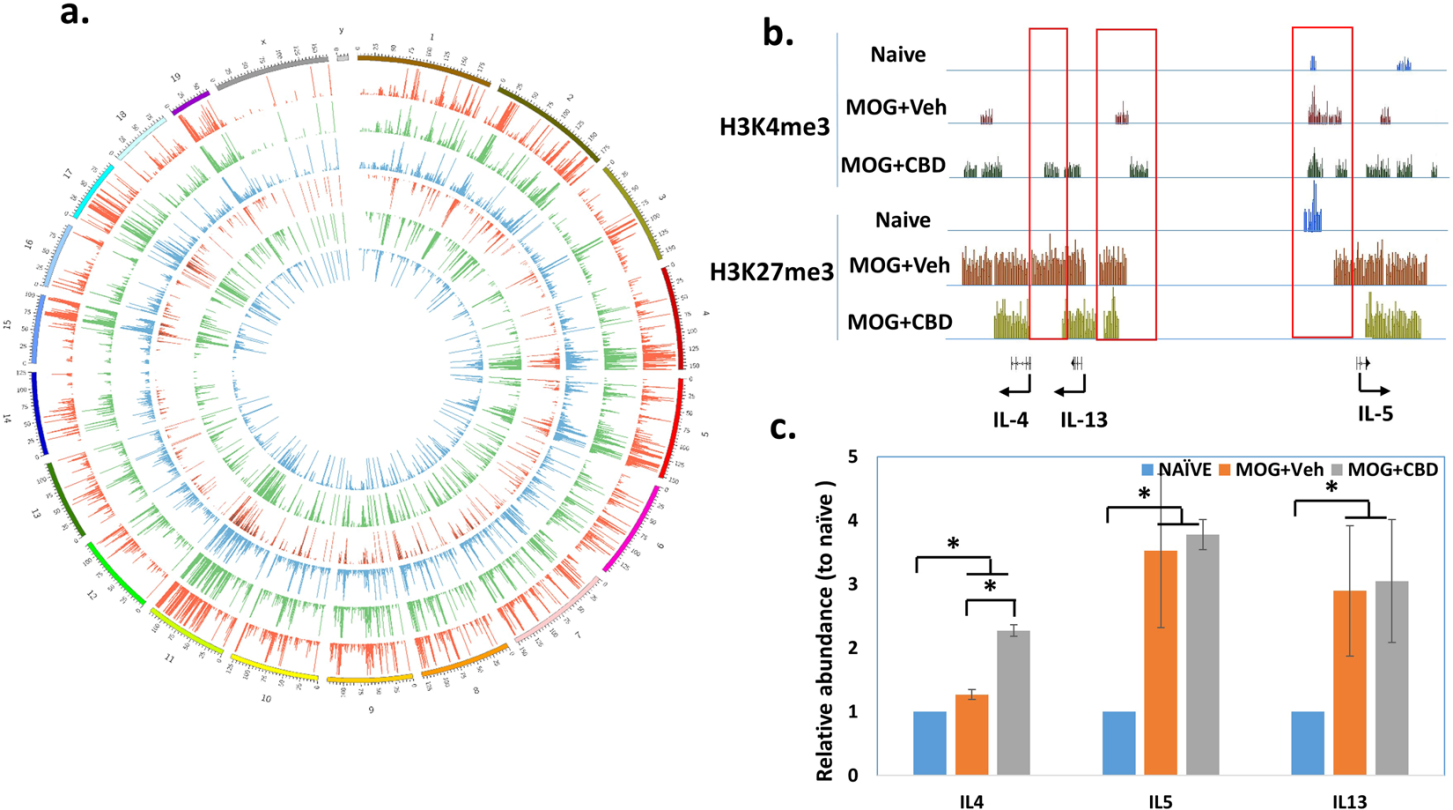
Histone methyaltion in splenic CD4+ T cells. CD4+ T cells from spleens of naïve, MOG + vehicle and MOG + CBD treated mice were isolated as described in the Materials and Methods. Histone methyaltion was determined by ChIP-seq. (**a**) Genome-wide histone methyaltion marks, H3K4me3 (outward histograms) and H3K27me3 (inward histograms) are presented using Circos plot. Blue: Naïve; Green: MOG + vehicle; Red: MOG + CBD. (**b**) H3K4me3 and H3K27me3 methylation levels in the genomic region of IL-4, IL-13 and IL-5. (**c**) Relavite expression level of IL-4, IL-13 and IL-5 determined by real time qPCR (The level in the naïve mice was set as 1).

MOG-induced EAE is a well-established mouse model of MS. Recently, we demonstrated that CBD was highly effective in attenuating EAE^6^. To determine whether CBD would affect histone modification in CBD-mediated regulation of T cell functions in this model, we examined two histone marks, H3K4me3 and H3K27me3. While many types of histone methylation have been shown to regulate gene expression, these two histone marks are the most well studied ones. H3K4me3 in the promoter region is associated with transcription activation and H3K27me3 is associated with transcription repression. The global histone methylation profile was examined by ChIP-seq in splenic CD4+ T cells from naïve, MOG + vehicle and MOG + CBD treated mice. The overall genome-wide H3K4me3 and H3K27me3 levels did not differ significantly among those 3 groups (Fig. 1a). However, the intensity and/or coverage of these histone marks in certain genomic regions was significantly altered by CBD in EAE mice. For example, the histone marks differed significantly in the genes of Th2 related cytokines such as IL-4, IL-5 and IL13 (Fig. 1b). Both marks showed lower levels of coverage in naïve cells when compared to MOG-activated cells, suggesting that histone methylation might be important for the expression of these cytokines in MOG activated T cells. Compared to vehicle treatment, CBD treatment led to a lower coverage of H3K27me3 but a higher level coverage of H3K4me3 in these genes, suggesting that CBD might cause an increase in the expression of these anti-inflammatory cytokines. The expression of IL-4, IL-5 and IL-13 in those 3 groups was further determined by real time PCR (Fig. 1c). When T cells were activated by MOG, the expression of IL-5 and IL-13 was significantly increased. CBD treatment tended to further increase their expressions although the difference was only significant for IL-4. This result indicated that besides H3K4me3 and H3K27me3, other histone/DNA modifications could also play important roles in the expression of these cytokines in EAE.

**Figure 2:**
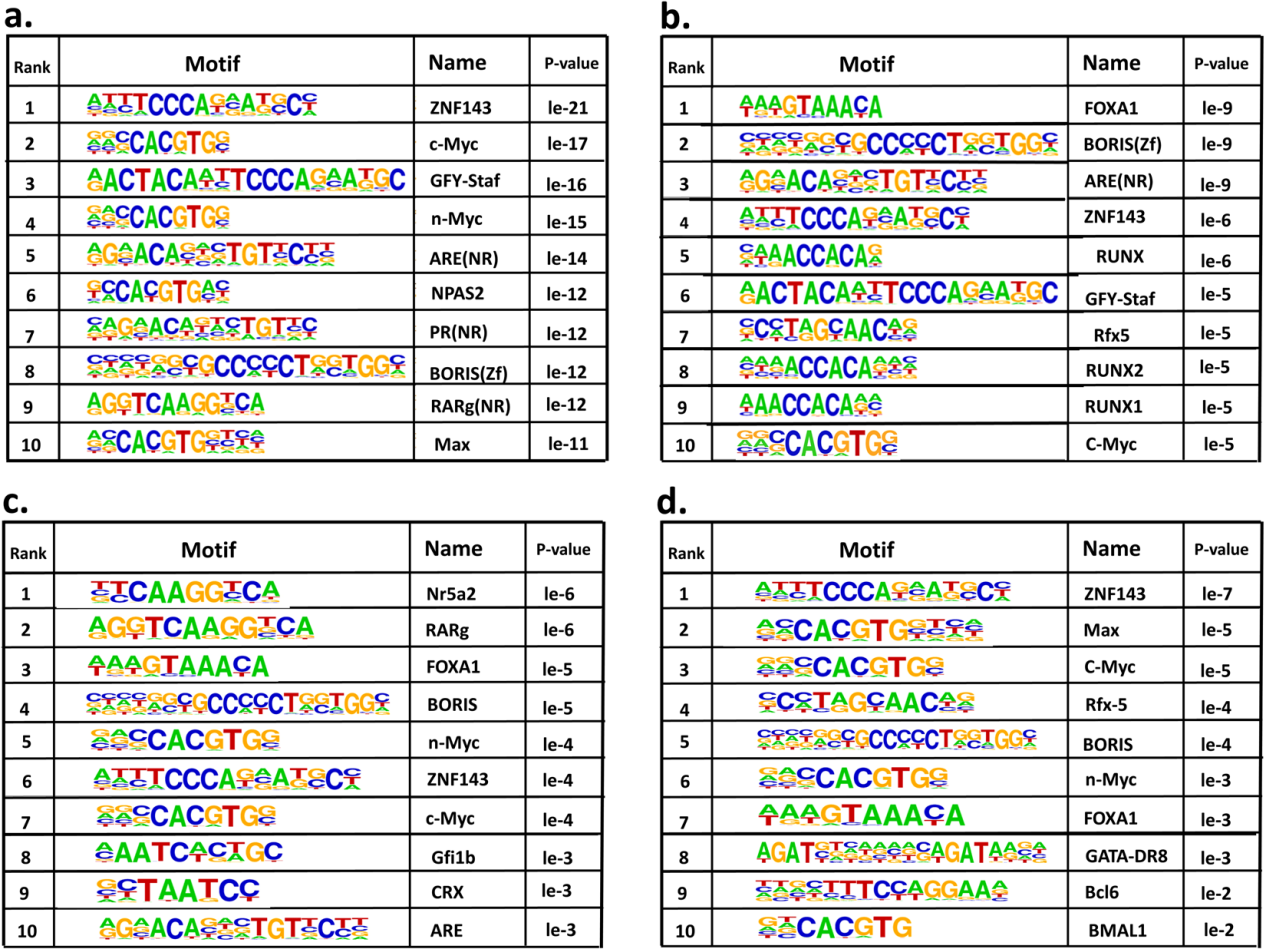
Enriched motifs with H3K4me3 and H3K27me3 marks in T cells. CD4+ T cells from spleens of naïve, MOG + vehicle and MOG + CBD treated mice were isolated as described in the Fig. 1 legend. Enrichment of motifs was identifed by HOMER sofwar using ChIP-seq data. Top 10 known transcription binding motifs are presented here. (**a**) Enriched motifs with H3K4me3 mark in MOG + vehich sample compared to Naïve sample; (**b**) Enriched motifs with H3K4me3 mark in MOG-sensitized T cells EAE mice treated with CBD compared to vehicle treatment; (**c**) Enriched motifs with H3K27me3 mark in MOG-sensitized T cells from EAE mice compared to Naïve; (**d**) Enriched motifs with H3K27me3 mark in MOG-sensitized T cells from EAE mice treated with CBD compared to vehicle treatment.

Because many transcription factors are involved in immune regulation, we further determined whether these 2 histone marks were differentially enriched in certain transcription factor binding motifs. By performing signal enrichment assay, we identified top 10 transcription factor binding motifs with enriched H3K4me3 or H3K27me3 signal after T cell activation by MOG (Fig. 2a,c). We also identified signal enriched motifs in CBD treated *vs*. vehicle treated cells (Fig. 2b,d). Among them were binding motifs of ZNF143, c-Myc, RUN family and FoxA1. ZNF143 and RUN family members are known to regulate DNA replication and cell cycle^20,21^, while FoxA1 is the factor for the development of a subset of Treg population^22^. Since histone modification affects transcription factor accessibility, the results suggest that genes whose expression is regulated by these transcription factors may be important for MOG-induced inflammation as well as CBD-induced anti-inflammation.

### CBD-mediated alteration in miRNA expression

**Figure 3:**
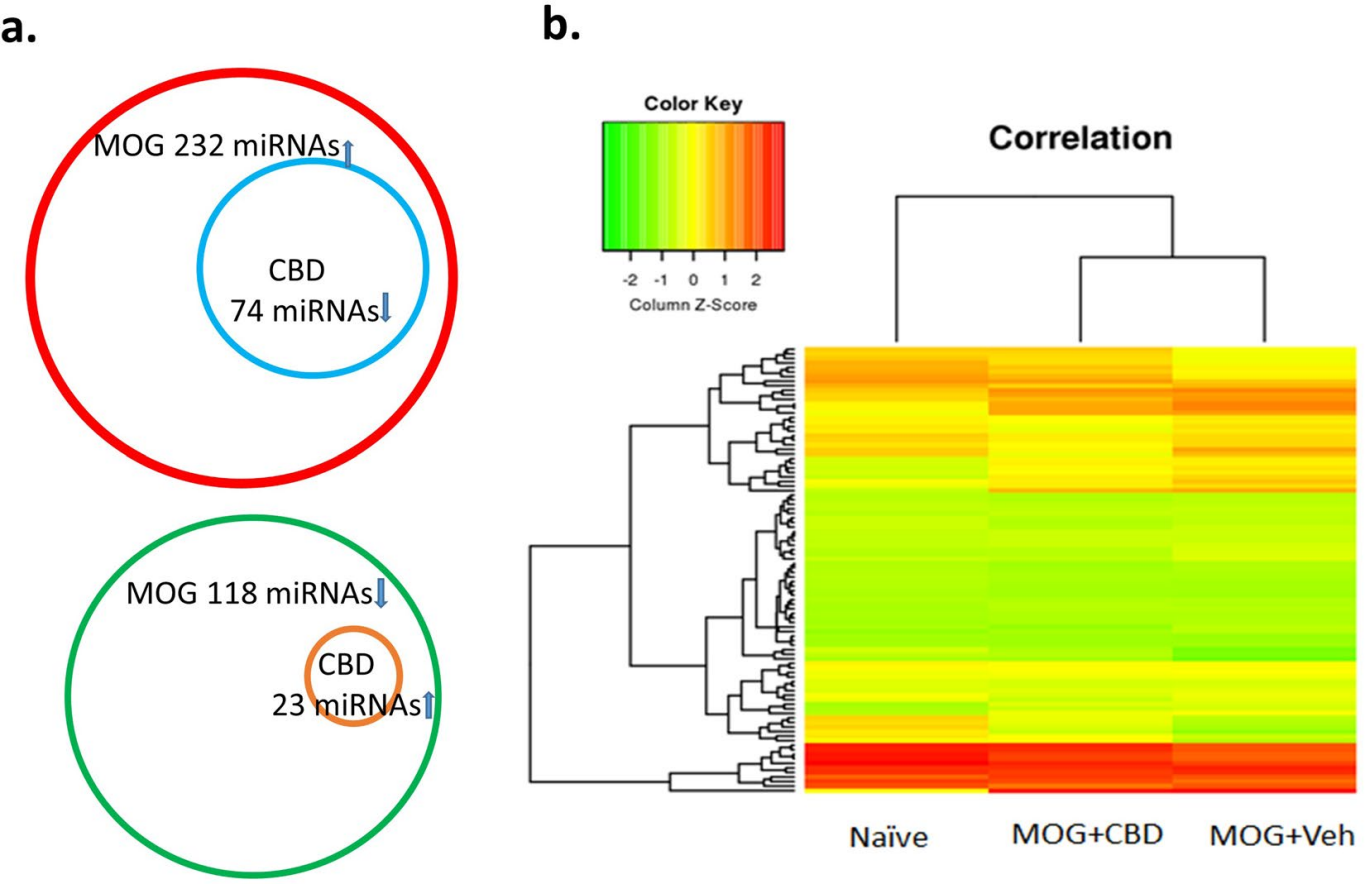
Expression of microRNA in splenic CD4+ T cells. CD4+ T cells from spleens of naïve, MOG + vehicle and MOG + CBD treated mice were isolated as described in the Fig. 1 legend. Expression of mature miRNAs in splenic CD4+ T cells from naïve mice and MOG-induced EAE mice treated with vehicle (MOG + Veh) or CBD (MOG + CBD) was determined by microarray. Linear fold change > 1.5 was considered as significant. (**a**) Number of MOG induced miRNAs (topper panel) and suppressed miRNAs (lower panel). CBD suppressed the expression of 74 MOG-induced miRNAs, and induced the expression of 23 MOG-suppressed miRNAs. (**b**) Heat map showing the expression levels of 1910 miRNAs in 3 groups of sample.

Inasmuch as miRNAs are known to play important role in the regulation of immune response, we compared the expression of mature miRNAs in splenic CD4+ T cells from naïve, MOG + vehicle and MOG + CBD groups by microarray. Among 1910 total mature miRNAs, 232 were significantly induced in MOG-activated T cells when compared to naïve T cells. Within those 232 miRNAs, 74 of them were suppressed after CBD treatment. On the other hand, MOG-treatment led to decreased expression of 118 miRNAs compared to naïve and only 23 of them whose expression was reversed by CBD treatment (see Supplementary Data). Figure 3 is the heat map of miRNA expression in these 3 groups.

### CBD-mediated alteration in transcriptome expression

**Figure 4:**
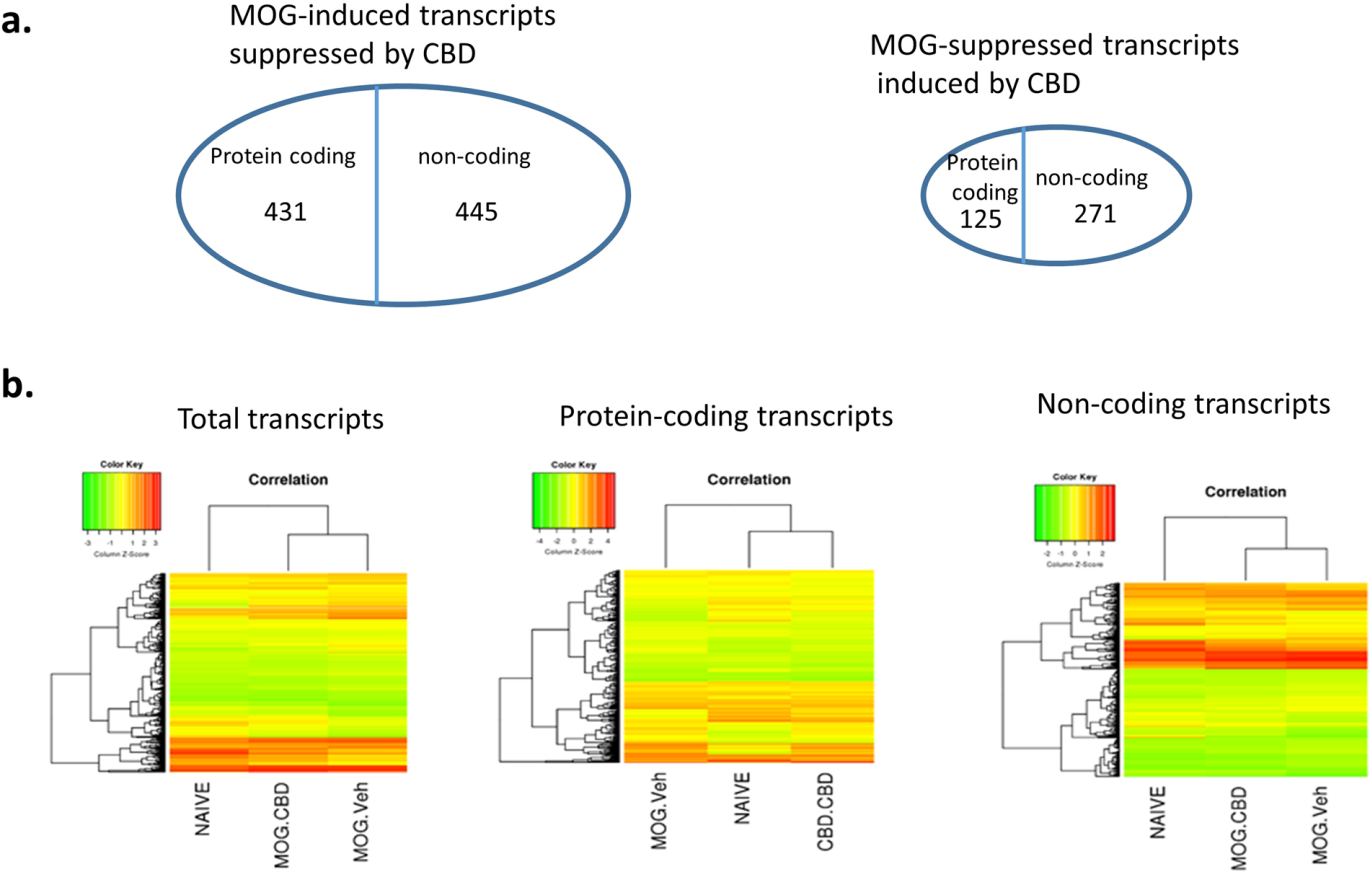
Transcriptome expression in splenic CD4+ T cells. CD4+ T cells from spleens of naïve, MOG + vehicle and MOG + CBD treated mice were isolated as described in the Fig. 1 legend. Gene expression in in splenic CD4+ T cells from maive, MOG + vehicle and MOG + CBD mice was determined by transcriptome microarray. (**a**) Number of protein coding and non-coding transcripts whose expression patterns were reversed by CBD treatment in EAE mice. (**b**) Heat map showing the expresseion levels of total transcripts as well as protein coding and non-coding transcripts.

**Figure 5:**
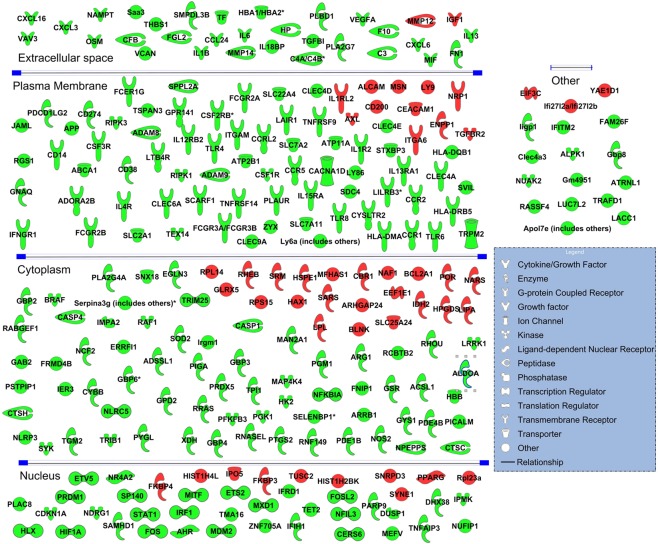
CBD regulated expression of immune related genes in splenic CD4+ T cells in EAE mice. Immune related genes whose expression pattern was reversed by CBD treatment in MOG-induced EAE mice are presented. Genes shown in green were induced in EAE mice (compared to naïve) but their expression was decreased by CBD treatment in EAE mice (compared to vehicle treatment). Genes shown in red were suppressed in EAE mice (compared to naïve) but their expressions were induced by CBD treatment in EAE mice (compared to vehicle treatment). The celluar localization of these gene products is also shown.

**Figure 6:**
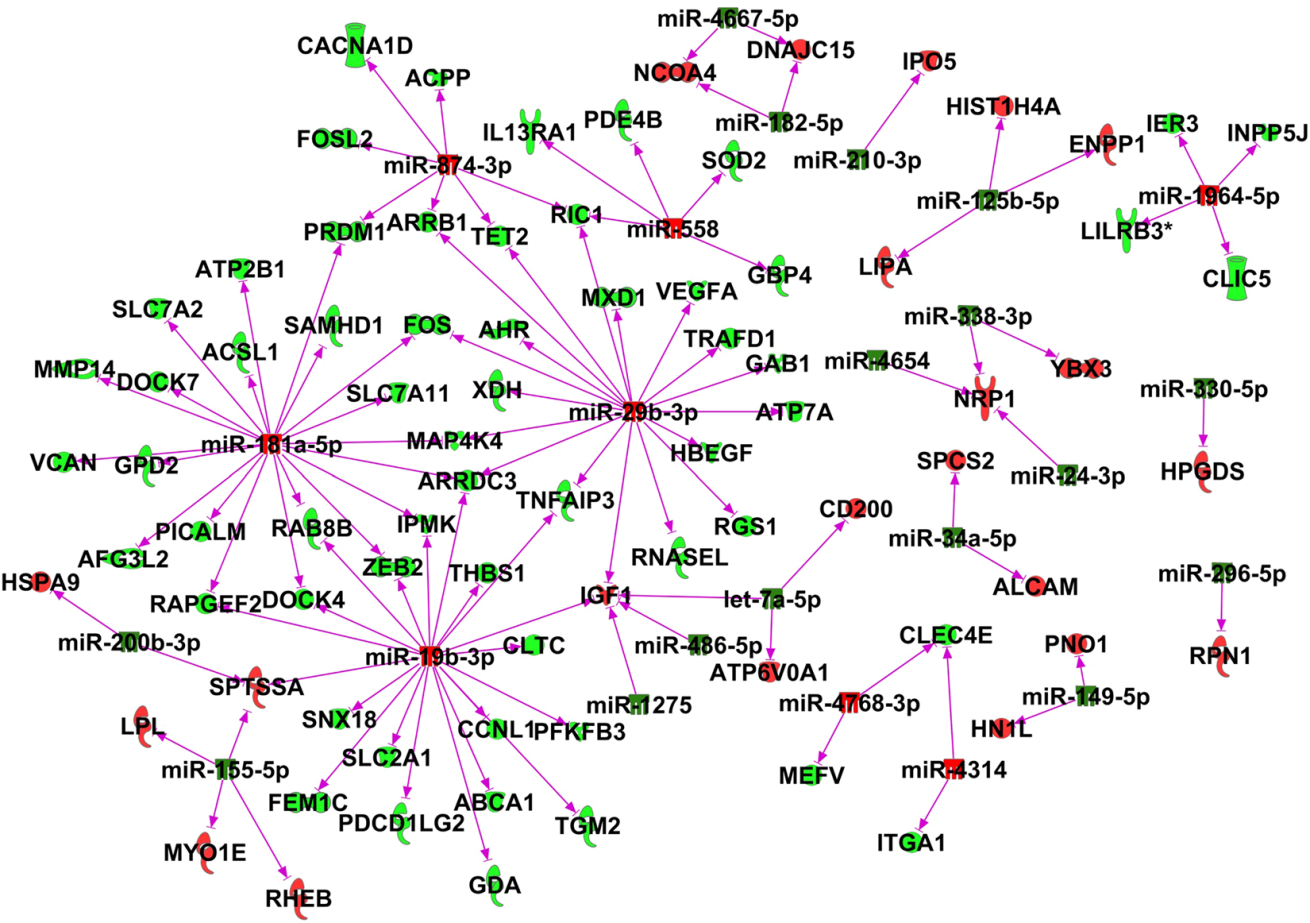
Expression pairing of differentially expressed genes and miRNAs in splenic CD4+ T cells in CBD treated EAE mice. Genes and miRNAs whose expression was significantly altered in MOG-induced EAE mice compared to naïve mice were used for expression pairing b IPA analysis (QIAGEN Inc., https://www.qiagenbioinformatics.com/products/ingenuitypathway-analysis). Those induced by CBD treatment in EAE mice are shown red and those suppressed by CBD are shown green.

We also used microarray to compare the transcriptome expression profile in CD4+ T cells isolated from these 3 groups of mice. MOG stimulation changed the expression of many transcripts. Among those altered transcripts, the expression pattern of 1272 transcripts was reversed by CBD treatment. CBD suppressed 876 MOG-induced transcripts, and induced 396 MOG-suppressed transcripts. Interestingly, majority of those altered transcripts were non-coding transcripts. CBD reversed the expression of 556 MOG altered protein coding transcripts and 716 non-coding transcripts (Fig. 4) (see Supplementary Data). Pathway analysis revealed that most altered protein coding transcripts were involved in cell cycle and immune response, which is consistent with the results that MOG increases while CBD inhibits T cell proliferation. The immune related genes as well as their cellular locations are presented in Fig. 5. We also paired altered miRNA from miRNA array with their potential targets from transcriptome array through the use of IPA (QIAGEN Inc., https://www.qiagenbioinformatics.com/products/ingenuity-pathway-analysis)^23^. Because miRNA usually suppress the expression of their targets, we only paired the protein coding genes and miRNAs that had opposite expression patterns after CBD treatment (Fig. 6).

### CBD mediated change in long non-coding RNA expression

**Figure 7:**
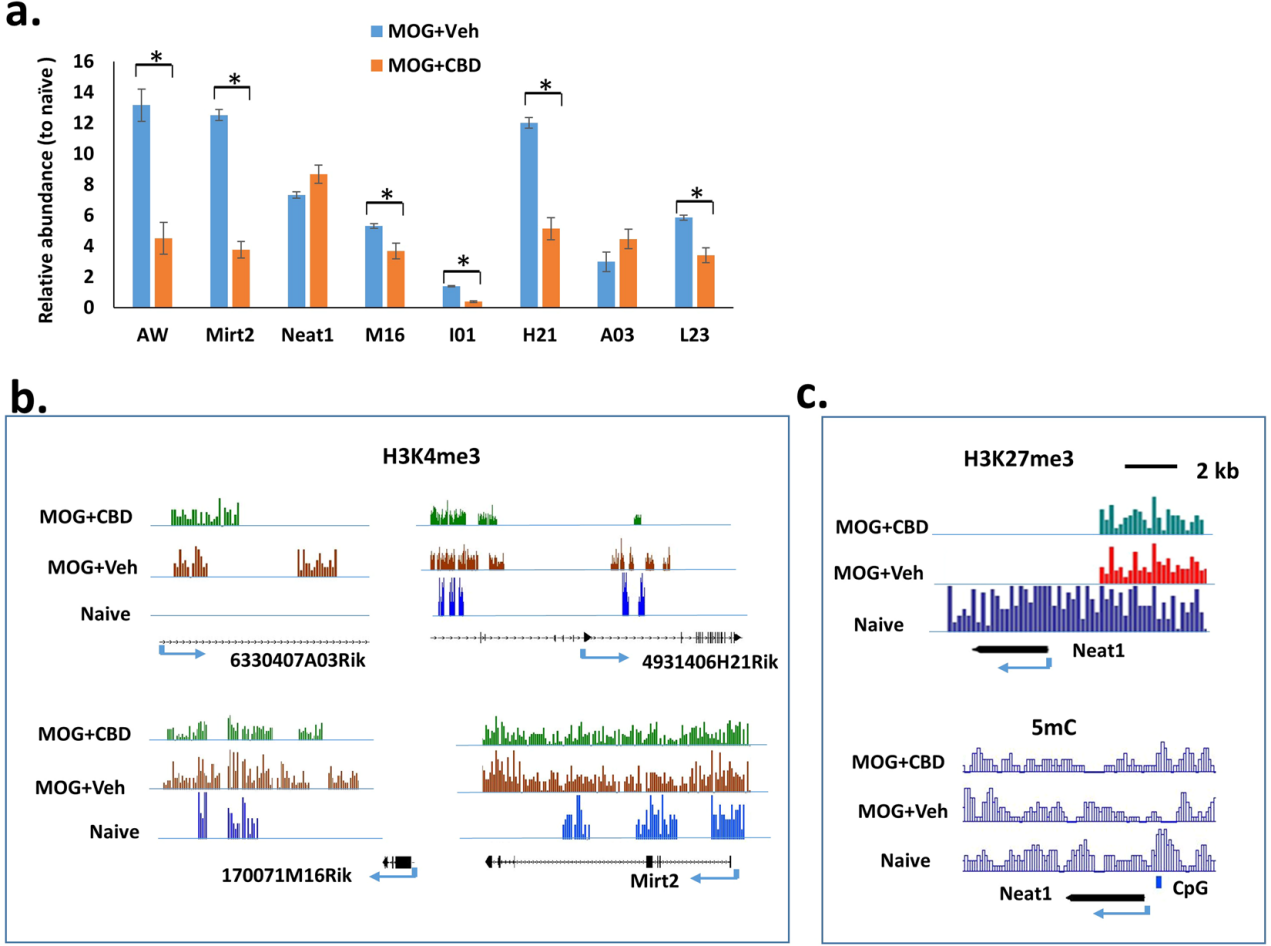
Expression and histone/DNA methylation of lncRNAs in splenic CD4+ T cells. The exprsession of select lncRNA in splenic CD4+ T cells was determined by real time PCR. The amount in cells from naïve mice was set as 1. (**a**) Relative exprssion level of lncRNAs in MOG-induced EAE mice treated with vehicle (MOG + veh) or CBD (MOG + CBD). (**b**) Histone H3K4me3 methylation in the selected lncRNA genes as determined by ChIP-seq. (**c**) Histone H3K27me3 methylation and 5mC in Neat1 gene.

Transcriptome array results showed that CBD altered the expression of many lncRNAs in splenic T cells in MOG-induced EAE mice. However, most of these lncRNAs are predicted ones and have not been validated experimentally. We selected some validated lncRNAs (AW112010 (AW), Mirt2, Neat1, 170071M16Rik (M16), 201011I01Rik (I01), 4931406H21Rik (H21), 6330407A03Rik (A03) and 9310230L23Rik (L23)), and their expressions in CD4+ T cells after MOG + vehicle and MOG + CBD treatment, were further confirmed by real time PCR (Fig. 7a). Although the functions of those lncRNAs are not known at the present time, the results indicated that those lncRNAs might play an important role in MOG-induced EAE development as well as CBD-mediated immune modulation. To determine whether their expressions correlated with histone/DNA modifications, we examined H3K4me3, H3K27me3 and 5mC in their genomic regions. The result suggested that the activation mark H3K4me3 was important for the expression of lncRNA A03, H21, M16 and Mirt2 (Fig. 7b). The suppressive marks might play an important role in the expression of Neat1, because in naïve T cells which had a low expression level, showed increased signals of H3K27me3 in its promoter and 5mC in its promoter CpG island (Fig. 7c). Overall, the patterns of histone H3K4me3 and H3K27me3 methylation as well as 5mC were consistent with the expression patterns of these lncRNAs.

## Discussion

CBD has been shown to have an anti-inflammatory effect in several animal models. While our previous studies demonstrated that CBD can induce MDSCs that are highly immunosuppressive and which upon adoptive transfer can attenuate EAE, the direct effect of CBD on T cells was not investigated. In this study, we examined the effects of CBD on global histone H3K4me3 and H3K27me3 methylation and gene expression in splenic CD4+ T cells from EAE mice. Despite the fact that MOG treatment dramatically increases transcription activity in T cells, the overall histone methylation levels do not differ significantly when compared to those in T cells from naïve mice. These results are similar to those from our previous study regarding the effect of THC on histone methylation in SEB activated lymphocytes^19^. These results indicate that MOG stimulation and CBD treatment may not alter global histone methylation/de-methylation activity. However, signal enrichment assay indicates that serval transcription binding motifs are differentially associated with histone methylation marks after CBD treatment in EAE mice. Among them is ZNF143 binding motifs. ZNF143 is a zinc finger protein which is critical for chromatin interaction at promoter area to regulate gene expression^24^. ZNF143 binds to promoters and is required for cell type specific chromatin interactions connecting promoters and regulatory elements^24^. Histone methylation such as H3K4me1 and H3K27ac in the ZNF143 binding sites has been linked to transcription activation^24^. In this study, we have found that ZNF143 binding motif is the top enriched motif associated H3K4me3 mark after MOG treatment, which suggests an enhanced activity of ZNF143 mediated transcription. Interestingly, ZNF143 binding motif is also the top enriched motif associated with the suppressive mark, H3K27me3, after CBD treatment in MOG stimulated cells, suggesting that both active and suppressive histone marks can modulate ZNF143 binding, which in turn, regulate gene expression.

FoxA1 binding motif is the top enriched motif with H3K4me3 mark after CBD treatment compared to the vehicle treatment in MOG stimulated cells. FoxA1 is a member of fork head family transcription factors and regulates the lineage-specific transcription^25^. It has been shown that FoxA1 binds to distinct regions within the genome with specific epigenetic modifications. FoxA1 binding sites have significant enrichment for transcription active marks, H3K4me1 and me2^25^. Our results suggest that CBD can also lead to the enrichment of H3K4me3 in the FoxA1 binding motif. Although FoxP3-expressing Treg cells have been shown to control inflammation in EAE model, studies have also suggested that other immunosuppressive T cells, such as FoxA1+ Treg cells, are involved in EAE and MS^22,26–28^. FoxA1+ Treg cells have been identified as IFN-β induced, linage-specific immunosuppressive cells, and IFN-β deletion leads to augmented symptoms of EAE^29^.

In our previous studies, we used RNA-seq to examine effect of cannabinoids such as THC on pri/pre-miRNA expression in SEB challenged lymphocytes^18^. Because the expression profile of precursor miRNAs may not correlate with their mature forms, we used microarray to determine mature miRNA expression in this study. Among those identified miRNA, some are known to regulate immune response. For example, miR-155 has been shown by us as well as by others to have pro-inflammatory property^30^. Some miRNAs such as miR-19, -210 and -223 that are up- regulated in T cells in MOG-induced EAE mice are also up-regulated in T cells from patients with MS^31^. On the other hand, few miRNAs such as miR-181c are down-regulated in EAE compared to naïve. miR-181c is also down-regulated in MS patients^31^. Furthermore, decreased expression of miR-181c in peripheral blood mononuclear cells from patients with myasthenia gravis correlates with elevated serum IL-7 and IL-17^32^. Knockdown of miR-181 has also been shown to enhance LPS-induced production of pro-inflammatory cytokines^33^, suggesting that miR-181c has an anti-inflammatory property. More importantly, the expression patterns of these miRNAs in EAE are reversed by CBD, indicating that modulating the expression of miRNA may constitute one of the mechanisms by which CBD exerts its anti-inflammatory roles in EAE and MS.

Long non coding RNA is another layer of gene expression regulation. We have found that many lncRNAs are induced by MOG treatment. However, the only lncRNA with known function is Neat1 (Nuclear-Enriched Autosomal Transcript 1). Neat1 is essential for the formation of paraspeckes in the nucleus and is a potent regulator in cell proliferation and differentiation^34,35^. Several studies have shown that aberrant expression of Neat1 is associated with various types of cancer, and its overexpression correlates with poor prognosis^36,37^. Overall, Neat1 seems to promote cell proliferation which is consistent with our results that MOG significantly induces Neat1 expression in T cells. Neat1 has also been shown to have a pro-inflammatory property. Neat1 expression is increased in Systemic Lupus Erythematosus and knockdown of Neat1 reduces cytokines such as IL-6 and CXCL10^38^. The expression of Neat1 is also induced by certain viral infections and facilitates the expression of antiviral cytokines such as IL-8^39^. Another MOG-induced lncRNA which is suppressed by CBD is Mirt2 (myocardial infarction-associated transcript 2). It is one of 2 lncRNAs that is up regulated in mice with myocardial infarction^40^. Further study has shown that Mirt2 may be involved in left ventricular remodeling^40^. A recent study shows that the expression of Mirt2 in macrophages is induced by LPS^41^. Furthermore, Mirt2 inhibits the activation of NF-kB and MAPK pathways to limit the expression of proinflammatory cytokines and thus it may serve as a negative regulator of inflammation^41^. Another lncRNA, AW112010, was initially identified as a transcript that might encode a small peptide but was classified as a non-coding RNA^42^. The role of this lncRNA is not known. One study shows that AW112010 is induced in microglia and astrocyte in CNS after virus infection, suggesting it may be involved in inflammation^43^.

In summary, the current study demonstrates that CBD modulates immune response through various mechanisms, from histone and DNA methylation to miRNA and lncRNA expression. The main purpose of this study is to identify potential regulatory elements in CBD-mediated immune modulation. Many candidates identified in this study have no known function and thus the current study provides new avenues to investigate their roles in regulating inflammation.

## Materials and Methods

### Induction of EAE and Cell Isolation

EAE was induced in female C57BL/6 J mice (8–10 weeks old) by subcutaneous injection of MOG_35–55_ (NeoMPS, San Diego, CA), as described^6,44,45^. Each mouse received 150 μg of MOG in 100 μl of complete Freund’s adjuvant with 100 μg of inactivated *Mycobacterium tuberculosis*. Mice also received 200 ng of pertussis toxin (Sigma) in 100 μl of PBS by intraperitoneal injection. Pertussis toxin was injected again 2 days after MOG injection. After MOG injection, mice received CBD (10 mg/kg of body weight) or the vehicle daily by intraperitoneal injection. All animal experiments were carried out in accordance with NIH guideline and approved by University of South Carolina IACUC (protocol #2363; approval data: May 31, 2017). After 7 days, spleens from the naïve control, MOG + vehicle and MOG + CBD (4–7 mice in each group) were collected and single cell suspension was prepared. CD4+ T cells were isolated using EasySep selection kit (StemCell Technologies, Vancouver, BC) according to the provided instructions. The antibody used was FITC-anti-mouse CD4 (BioLegend, San Diego, CA). Isolated cells were cultured in complete RPMI1640 medium. For the MOG + vehicle group, 30 μg/ml of MOG was added to the medium. For the MOG + CBD group, 30 μg/ml of MOG and 10 μM of CBD were added to the medium. Cells were harvested after 48 hr of culturing.

### ChIP-seq

ChIP-seq was performed as described previously^19,46^. Briefly, crosslinked chromatin was fragmented by Micrococcal nuclease digestion and immunoprecipitated with anti-H3K4me3 or anti-H3K27me3 antibodies (Abcam, Cambridge, MA). After ChIP, the crosslinking was reversed and DNA fragments were purified. The sequencing library was constructed using Illumina’s TruSeq sample preparation kit according to the provided instruction. The samples before immunoprecipitation served as input controls. Sequencing was performed using Illumina Nextseq 500 sequencer.

### miRNA and transcriptome microarray

RNA was purified by RNeasy and miRNeasy kit (Qiagen,). For miRNA quantification by Affymetrix GeneChip miRNA array, mature miRNAs were labeled by FlashTag Biotin HSR RNA labeling kit. For transcriptome expression analysis by GeneChip Whole Transcript Expression Array, samples were amplified and labeled using GeneChip WT PLUS reagent kit. Microarray hybridization, wash and staining were performed using the Fluidics Station 450. All procedures were carried out according to the protocols provided by Affymetrix. Data were analyzed by Affymetrix Expression Console software. Fold changes >1.5 were considered as significant.

### Quantitative Real-time PCR

Total RNA including miRNA was reverse transcribed by miScript II RT kit (Qiagen). mRNA expression was measured by SYBR Green PCR kit and mature miRNA was quantified by miScript Primer Assays (Qiagen). *Snord96a* and *Gapdh* were used as the internal controls for miRNA and mRNA, respectively. The amount in the naïve CD4+ T cells was set as 1.

### Data analysis

ChIP-seq data were analyzed as described previously^19,46^. The signals were visualized in Integrated Genome Browser (www.bioviz.org) using mouse mm9 genome build as the reference. The genome-wide histone methylation map was generated by the Circos plot^47^. The signal enriched motifs were identified using HOMER motif analysis software^48^. The heatmaps were generated by the R program. Ingenuity Pathway Analysis (Qiagen) was used for functional analysis and pairing of miRNA and mRNA. Statistical analysis was performed using T test (For animal experiments, each group had 4 to 7 mice. For qPCR, n = 3). Significance was determined as p < 0.05 (denoted by *).

## Supplementary information

Dataset1
